# Microenvironmental Variations After Blood-Brain Barrier Breakdown in Traumatic Brain Injury

**DOI:** 10.3389/fnmol.2021.750810

**Published:** 2021-11-26

**Authors:** Yue Hu, Weiwei Tao

**Affiliations:** ^1^School of Chinese Medicine, School of Integrated Chinese and Western Medicine, Nanjing University of Chinese Medicine, Nanjing, China; ^2^Jiangsu Collaborative Innovation Center of Chinese Medicinal Resources Industrialization, National and Local Collaborative Engineering Center of Chinese Medicinal Resources Industrialization and Formulae Innovative Medicine, Nanjing University of Chinese Medicine, Nanjing, China

**Keywords:** traumatic brain injury, blood-brain barrier, microenvironment, edema, inflammation, toxic substances, recovery

## Abstract

Traumatic brain injury (TBI) is linked to several pathologies. The blood-brain barrier (BBB) breakdown is considered to be one of the initial changes. Further, the microenvironmental alteration following TBI-induced BBB breakdown can be multi-scaled, constant, and dramatic. The microenvironmental variations after disruption of BBB includes several pathological changes, such as cerebral blood flow (CBF) alteration, brain edema, cerebral metabolism imbalances, and accumulation of inflammatory molecules. The modulation of the microenvironment presents attractive targets for TBI recovery, such as reducing toxic substances, inhibiting inflammation, and promoting neurogenesis. Herein, we briefly review the pathological alterations of the microenvironmental changes following BBB breakdown and outline potential interventions for TBI recovery based on microenvironmental modulation.

## Introduction

Traumatic brain injury (TBI) is a critical public health problem in many areas worldwide, especially in the developed countries (Hydera et al., 2007; Corrigan et al., 2010; Roozenbeek et al., 2013). This injury has both short- and long-term effects on prognosis, such as TBI-associated disabilities, amnesia, depression, and other related physical or mental disorders (Dixon, 2017). The studies have found out that not only severe TBI, but also mild TBI result in long-term sequelae and psychological morbidity (Levin and Diaz-Arrastia, 2015). Despite the well-developed medical management of TBI in the United States and other countries (Stonesifer, 2008; Coronado et al., 2012), many of the survivors of TBI do not fully recover and left permanent sequela. Thus, novel perspective of pathophysiologic mechanism for TBI and the therapeutic targets are desperately needed.

The microenvironment around neurons and other cells in brain parenchyma consists of elements that greatly influence the conditions around a cell or a cell cluster, and these elements may play a direct or indirect role in affecting cell behavior biophysically or biochemically (Charles et al., 2011). Since TBI is a complex and heterogeneous disease, microenvironment in the lesion areas following TBI may changes multi-scaled, constantly and dramatically (Hemphill et al., 2015). The cell–cell and cell–matrix interactions are greatly regulated by the molecules or factors which consist in microenvironment, suggesting that the microenvironmental changes in brain play an essential role in brain injury and remodeling after TBI (Kan et al., 2012; Teschemacher et al., 2015).

Because of blood-brain barrier (BBB), most compounds from blood to brain were impeded (Daneman, 2012; Zhao et al., 2015). Thus, BBB is one of the most important sites for the control of the central nerve system (CNS) microenvironment and homeostasis (Ballabh et al., 2004; Lampron et al., 2013). At present, many researchers show great interest in the association of brain microvessels, pericytes, astrocytes, and neurons to form functional “neurovascular units” (NVU), which contribute to neurovascular coupling (McCarty, 2009; Chen et al., 2014). In addition, the BBB is the most important structure of NVU not only anatomically but also physiologically (Muoio et al., 2014; Price et al., 2016). When TBI occurred, the BBB breakdown frequently follows, and might lead to the signaling cascades and complex interactions between the pathological processes within the NVU (Korn et al., 2005; Tomkins et al., 2008), such as edema, neuroinflammation, and cell death (Shlosberg et al., 2010). These processes are closely associated with the microenvironmental changes in the damaged brain (Kan et al., 2012).

In this review, we briefly discussed the pathological alteration of TBI after BBB breakdown and the microenvironmental changes related to BBB dysfunction, e.g., the cerebral metabolic changes, cerebral blood flow (CBF), toxic molecules accumulation, inflammation, and edema. In addition, we outlined the potential intervention schemes that target BBB-related microenvironment balance, homeostasis, and improvement for post-TBI recovery.

## Traumatic Brain Injury and Blood-Brain Barrier Dysfunction

### The Structure and Function of Blood-Brain Barrier

Since first observed by Paul Ehrlich in 1885, until recent decades, basically, the BBB has well-known as a complex, dynamic, adaptable structure to prevent the uncontrolled leakage of substances from the blood into the brain. Herein, we briefly overview the structure and function of BBB.

**FIGURE 1:**
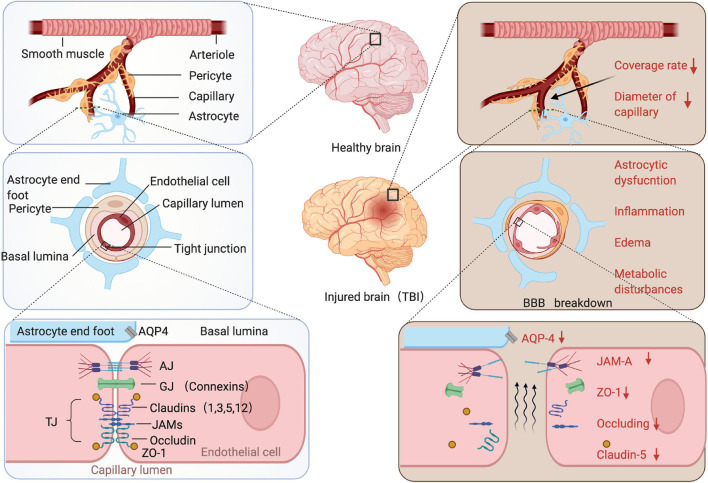
The stepwise amplified structure of the BBB of the healthy or injured brain. Arterioles branch off into capillaries, and capillaries are covered by pericytes and astrocytes end-feet. The pericytes and endothelium share a common basement membrane and connect with each other with several transmembrane junctional proteins. After traumatic brain injury (TBI), coverage rate of the pericytes dramatically decreased and diameter of capillary reduced, junction proteins were downregulated. There are several pathological changes occur following TBI, e.g., astrocytic dysfunction, inflammation, edema, and metabolic disturbance.

Anatomically, the elements compose the BBB are the endothelial cells, astrocyte end-feet, pericytes, and the basement membranes (BM) (Figure 1, left panel): (1) For endothelial cells, they are the central component of the BBB, connected with each other through the tight junction (TJ), adheres junction (AJ), and gap junction (GJ) proteins (Liebner et al., 2018; Sharif et al., 2018). TJs composed of at least three major transmembrane proteins, such as claudin, occludin, and junctional adhesion molecules (JAMs) (Furuse et al., 1999; Balda et al., 2000; Mankertz et al., 2002; Wolburg and Lippoldt, 2002). These proteins form an impermeable barrier to fluid. In addition, many cytoplasmic proteins involved in TJ formation include zonula occludens proteins (ZO-1, ZO-2, and ZO-3), cingulin, 7H6, and so on (Matter and Balda, 2003; Tepass and Harris, 2007; Peglion et al., 2014). (2) The end-feet of astrocyte tightly sheath the vessel wall and the loss of contact between the end-feet and blood vessels also leads to a loss of TJ (Willis et al., 2004; Watkins et al., 2014). The astrocytes promote the BBB creation and maintenance by the release of various secreted factors which may be important to contribute to vessel stabilization and junctional proteins regulation (Janzer and Raff, 1987; Wolburg and Lippoldt, 2002; Lee et al., 2003; Alvarez et al., 2013; Broux et al., 2015). In addition, the astrocytes produce the biochemical enzymes and regulate blood flow which is important for BBB maintenance (Wolburg-Buchholz et al., 2009; MacVicar and Newman, 2015). (3) The pericytes share a basement membrane with endothelial cell (Attwell et al., 2016), and anchored to the basement membrane *via* integrins (Armulik et al., 2010). They confirmed to play the essential roles in maintaining BBB integrity (Daneman et al., 2010; Armulik et al., 2011), regulating capillary diameter, and CBF (Yemisci et al., 2009; Fernández-Kletta et al., 2010; Hall et al., 2014; Sweeney et al., 2016), promoting angiogenesis (Winkler et al., 2011) and phagocytosing toxic metabolites (Hartmann et al., 2015). Moreover, signaling between the astrocytes and pericytes exerts significant impact on BBB integrity (Yao et al., 2014; Mishra et al., 2016). (4) The BM abound all the kinds of cells mainly consist of type IV collagens, laminins, nidogen, and HSPGs also vital for BBB structural integrity. Because access of the molecules and cells to the CNS parenchyma requires not only crossing the endothelial cell, but traversing both the layers of BM (Banerjee et al., 2016).

For function of BBB, in the physical condition, BBB are permeable to O_2_ and CO_2_ as well as other gaseous molecules, such as helium, N_2_, and many gaseous anesthetics. In addition, BBB is also permeable to water and lipid soluble. However, transfer of some molecules, especially the macromolecules through BBB are limited, it seems that the regulation of macromolecules is more complicated and usually mediated with transporters (Pardridge, 2005; Obermeier et al., 2013; Serlin et al., 2015). BBB permeability contains two aspects: (1) the ions and other small molecules cross the BBB by paracellular diffusion through the junctional complex or by the transcellular pathway across the cells. However, in some circumstances, the tight junctions may limit the paracellular flux of hydrophilic molecules across the BBB (Cancilla and DeBault, 1983; Simard and Nedergaard, 2004; Jeong et al., 2006). (2) For the macromolecules, accumulating evidence suggests that the large molecular weight serum proteins infiltration though a dysfunctional BBB carries a potential risk for pathological outcomes (Tajes et al., 2014). Thus, nearly 98% of all these molecules are not freely transported across the BBB (Pardridge, 2005). The delivery of large molecules, such as the proteins and peptides are mainly regulated by adsorptive-mediated transcytosis (AMT) and receptor-mediated transcytosis (RMT) (Dogrukol-Ak et al., 2009). Both of these processes result in passage across the BBB.

A new concept is that the BBB changes from “barrier” to “interface,” which means this structure is not only a substantial barrier for drug delivery to the brain but also a complex, dynamic interface that adapts to the needs of the CNS (Banks, 2016). BBB itself is now considered to be a therapeutic target for CNS disease and is often more accessible to the manipulation than the cells that it protects (Cho et al., 2017).

### Blood-Brain Barrier Breakdown Following Traumatic Brain Injury

Under the physiological conditions, the BBB acts as a barrier that impairs the access of molecules and immune cells, such as monocytes, lymphocytes, and other leukocytes. However, BBB can easily breakdown in many neurological diseases, such as brain trauma, stroke, as well as other neurodegenerative disorders, such as Alzheimer’s disease and Parkinson’s disease (Kortekaas et al., 2005; Bowman et al., 2007; Zlokovic, 2008).

In general, TBI can be divided into two phases: primary and secondary injury (Hawryluk and Manley, 2015; Hay et al., 2015). The primary injuries are the result of mechanical forces causing compressive and shearing injuries, the secondary injuries are the consequence of subsequent damages, such as hypoxia, inflammation, and metabolic disturbances (Sahuquillo et al., 2001; Shlosberg et al., 2010; Lozano et al., 2015). Both the animal model and substantial clinical data indicated that BBB disruption frequently follows brain trauma and can last from several days to weeks (Tomkins et al., 2001; Korn et al., 2005). In the focal controlled cortex impact CCI animal model, the severe force delivered to the brain directly cause BBB disruption (Barzo et al., 1996; Esen et al., 2003), which is called as primary BBB damage. Following the infliction of a focal head impact, the small blood vessels often incur a concomitant shear injury, which lead to the impairments in the regulation of the BBB, CBF, and metabolic processes (Rodriguez-Baeza et al., 2003; Akbik et al., 2016). During the secondary phase, the abnormalities in the BBB can arise the abnormal brain activity, astrocytic dysfunction (Wolburg-Buchholz et al., 2009; Heinemann et al., 2012), inflammatory responses (Harting et al., 2008; Plesnila, 2016), brain edema (Unterberg et al., 2004), and metabolic disturbances (Alluri et al., 2015).

For BBB structures damage, an inevitable consequence of BBB breakdown is an increase in the permeability of the damaged endothelium (Bhowmick et al., 2019). Following TBI, the endothelium-associated tight junction proteins JAM-A, ZO-1, occludin, and claudin-5 were down-regulated indicating acute TBI-associated tight junction protein disruption (Evran et al., 2020; Sivandzade et al., 2020; Kempuraj et al., 2021). The studies showed that after animal TBI model, as many as 40% of the pericytes loss the contact of basement membrane within the first hours of the injury (Dore-Duffy et al., 2000). Then, the diameter of the arteriolar and capillary was reduced at a later time point following TBI (Prager et al., 2019). For astrocyte end-feet, AQP4 proteins are expressed abundantly on the perivascular end-foot membranes and astrocytic membranes in a polarized pattern, which mainly contribute to edema that evolves after TBI. The studies demonstrated that expression of AQP4 on the perivascular end-foot membrane reduced rapidly following TBI (Lu et al., 2020; Ma et al., 2021; Figure 1, right panel). In chronic phase, the mural cells (pericytes and smooth muscle cells) can be degenerated up to 12 months post injury, causing the alterations in tau uptake may further contribute to tau deposition in the brain (Ojo et al., 2021).

It seems that following TBI-induced BBB breakdown, together with the damage of BBB structure, microenvironmental homeostasis is quickly destructed. The imbalance of microenvironment may lead to further damage to BBB, on the other side, targeting some novel factors to improve the brain microenvironment may provide a potential approach to TBI recovery.

## Microenvironmental Changes Following Traumatic Brain Injury-Induced Blood-Brain Barrier Breakdown

**FIGURE 2:**
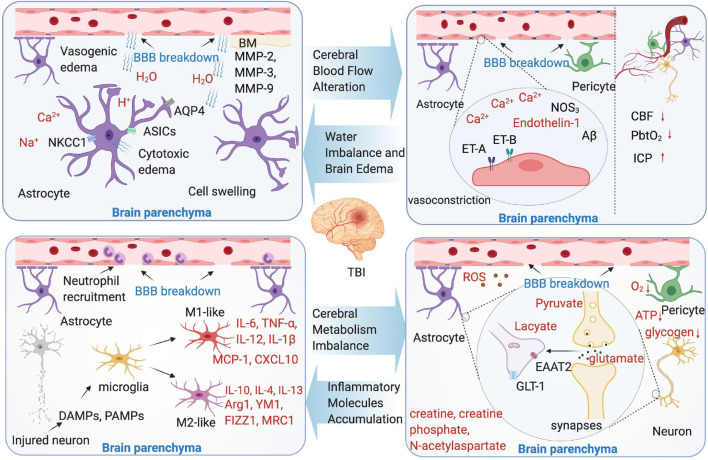
Microenvironment changes following TBI-induced blood-brain barrier (BBB) breakdown. Four aspects were shown as cerebral blood flow (CBF) alteration, water imbalance and brain edema, cerebral metabolism imbalance, and inflammatory molecules accumulation. The text marked red in the picture indicate the substance in brain parenchyma microenvironment.

Although the underlying molecular changes in the microenvironment following TBI are not completely clear, with the development of microdialysis, angiography, imaging, and other techniques, our understanding of the microenvironmental changes after TBI become deeper. This section discusses the new perspective on the microenvironmental changes following TBI-induced BBB breakdown (Figure 2).

### Cerebral Blood Flow Alteration

It is already clear that both O_2_ and glucose are delivered to the neurons by CBF and are transported across the BBB (Moskowitz et al., 2010). CBF regulation involves complicated mechanism and contains many types of cells, such as pericyte and astrocyte (Hall et al., 2014; Hill et al., 2015; MacVicar and Newman, 2015; Kisler et al., 2017). Proper structural and functional BBB connectivity, synaptic activity, and information processing all requires precise regulation of CBF (Attwell et al., 2010). In TBI, the measurement of CBF can be invasive or non-invasive (Rostami et al., 2014), the markers of CBF, such as brain tissue oxygenation (PbtO_2_), Jugular venous bulb oximetry (SjvO_2_), ICP, and CPP, each has inherent limitations (Akbik et al., 2016).

Numerous findings from the animal TBI models have linked the endothelium cells to decreased CBF and poor outcome following brain injury. In brain vascular system, the endothelium cells, which is the main structure of BBB, play a key role to maintain vascular integrity and microenvironmental homeostasis (Graves and Kreipke, 2015). Endothelin-mediated vasoconstriction that decreases arterial luminal areas is the main reason of CBF reduction in TBI. The main mechanism is that vasoconstriction through the synthesis of endothelin-1 or upregulate endothelin receptors A and B (Faraci and Breese, 1993; Steiner et al., 2004; Kallakuri et al., 2010; Schwarzmaier et al., 2015b). In addition, in the mild to moderate TBI model, mitochondrial Ca^2+^ uptake improves CBF, and the intervention of this pathway may reduce behavioral deficit (Murugan et al., 2016). The pericytes and astrocyte end-feet swelling are found to contribute to CBF regulation (Ostergaard et al., 2014). Astrocytic end-feet swelling has been observed as early as 1 h after TBI (Dietrich et al., 1994), and lasts until 11 days after the initial injury (Bullock et al., 1991), which cause compression of the capillary lumen that negatively affect CBF in the injured brain. The pericytes are involved in the regulation of capillary diameter to affect CBF. After brain insult, the pericytes leave their pericapillary location within the first hour (Dore-Duffy et al., 2000), and decline in the acute phase. However, in the trauma zone, the pericytes increase days after the initial injury (Zehendner et al., 2015). It seems that the brain trauma causes a biphasic response of pericytes in the early phase of brain trauma. Loss of pericytes or the impairment of pericyte-endothelium interaction increases the BBB permeability, facilitates the formation of brain edema, and decreases the CBF in the surrounding parenchyma (Bhowmick et al., 2019). Additionally, the variants of some genes are confirmed to be related with CBF alteration in an animal TBI model. These genes include NOS3 and Aβ (Abrahamson et al., 2013).

### Water Imbalance and Brain Edema

Following the primary injury of TBI, the structural and functional integrity of the BBB is disrupted, the alterations in blood flow lead to the hypoxic conditions in tissue with the activation of proteases, initiation of inflammatory pathways, generation of toxic substance, and production of reactive oxygen species (ROS), which are described previously, leading to brain edema. This edema is the result of BBB injury and can further cause tissue damage, it can be mainly classified into two types: vasogenic and cytotoxic (Unterberg et al., 2004; Lukaszewicz et al., 2011; Jha et al., 2019).

Briefly, the definition of vasogenic edema is that the water moves from the vasculature to the extracellular space, results in brain water content increase, tissue swelling, and ICP increase. Thus, the vasogenic edema from BBB opening considered to be the main contributor of the injury (Reulen et al., 1977). By using a two-photon microscopy and *in vivo* 3D deep-brain imaging, TBI induces vasogenic brain edema that is identified from capillaries, venules, and arterioles (Schwarzmaier et al., 2015a). Moreover, the development of vasogenic edema showed a biphasic pattern, peaking 4 and 48–72 h after TBI (Hu et al., 2021). Cytotoxic edema is characterized by the sustained intracellular water accumulation, this type of edema usually associated with a failure of the ATP-dependent Na^+^/K^+^-pumps, which further lead to the cellular ionic content increase and influx of water into the neuronal and other cells (Shapira et al., 1993). In contrast to vasogenic brain edema, cytotoxic edema with no change in tissue water content or volume and independently of the BBB integrity. Osmotic brain edema develops with osmotic gradient, and the imbalances between the blood and tissue cause cell swelling as cytotoxic edema does (Katayama and Kawamata, 2003; Unterberg et al., 2004). Additionally, numerous mediators are identified that are involved in the process of brain edema, for instance, aquaporins (AQPs), matrix metalloproteinases (MMPs), and vasoactive agents following BBB breakdown (Ke et al., 2002; Higashida et al., 2011; Blixt et al., 2015). The AQP4 is associated with the cytotoxic edema (Haj-Yasein et al., 2011), however, the opinions are controversial: the inhibition of AQP4 expression is identified associated with the brain edema reduction (Fazzina et al., 2010; Keisuke et al., 2010), however, conversely, in the AQP4 knockout animals, vasogenic edema was exacerbated after cold lesion injury, identified that AQP4 may have the function to reduce vasogenic edema (Papadopoulos et al., 2004). Other studies focus on target AQP4 to treat brain edema following TBI-induced BBB breakdown, such as oloxamer-188, edaravone, and nerve growth factor (Kikuchi et al., 2009; Bao et al., 2012; Lv et al., 2013). The MMPs are zinc-dependent endopeptidases involved in the formation of BBB. The MMPs, mainly include MMP-2, MMP-3, and MMP-9, all upregulated in the TBI animal models (Asahi et al., 2001; Falo et al., 2006; Alluri et al., 2016). The MMPs can cause BBB breakdown and further vasogenic edema, especially MMP-9. The result of a recent study shows that, in MMP-9 knock-out mice, BBB disruption was attenuated compared with the wild type mice (Asahi et al., 2001).

### Cerebral Metabolism Imbalance

It is well-known that the brain undergoes a metabolic crisis after TBI, especially after BBB breakdown. As a consequence of extracellular and intracellular ionic imbalance following neuronal activation, energy production has to take place (Lin et al., 2010; Mishra et al., 2011; Lovatt et al., 2012). Usually, BBB breakdown causes a mismatch between energy demand and supply, and the tissue metabolism is regionally heterogeneous following TBI (Buxton, 2010; O’Phelan et al., 2013; Brooks and Martin, 2014). With the CBF breakdown and limited oxygen delivery, the ionic and cellular homeostasis are destroyed, resulting in intracellular calcium flux, further mitochondrial dysfunction (Giza and Hovda, 2014). In the very early phases, the oxidative metabolism may occur, it can be measured by microdialysis and MR spectroscopy imaging (Alves et al., 2003; Belli et al., 2008). The initial oxidative metabolism increases the glucose uptake in a very short period of time, however, in long term, it worsens the energy crisis of TBI. With the metabolic pathways change, the glucose metabolic rates reduce due to the breakdown of ATP-dependent pumps/transporters, at the same time, other metabolic product changes as well, such as creatine, creatine phosphate, and N-acetylaspartate (Signoretti et al., 2009). Increasingly, the lactate pyruvate ratio, which reflects impairment of hypoxic episode or cellular respiration is dramatically changed. As consequence of anaerobic metabolism and glycolysis, the amounts of lactate increased, a study by Bouzat et al. (2014) showed that exogenous systemic lactate was utilized by the injured human brain as a preferential energy substrate in TBI. This study suggests that hypertonic lactate therapy has beneficial cerebral metabolic and hemodynamic effects after TBI.

The cytotoxic molecules are released, such as excitatory amino acids which can cause damage to the brain. In general, glutamate, which is taken up by the astrocytes, largely by excitatory amino acid transporter 2 (EAAT2) or glutamate transporter-1 (GLT-1), is considered to be a main contributor to cellular apoptosis (Jansson and Akerman, 2014; Guerriero et al., 2015). In TBI, glutamate increase is among the first events to occur post-injury, and results in destroying the astrocyte function and increase BBB permeability (Obrenovitch and Urenjak, 1997; Guerriero et al., 2015). Measured by cerebral microdialysis, the glutamate levels, not only in brain, but also in blood, are confirmed to correlated with the mortality rate and long-term functional outcome in TBI clinical practice (Chamoun et al., 2010; Quintard et al., 2015). In an animal CCI model, glutamate signaling is significantly increased in the injured cortex (Cantu et al., 2015), another study by Goodrich demonstrated that GLT-1 expression is depressed, which means more glutamate gathered (Goodrich et al., 2013).

By using two-photon microscopy, tissue oxygenation, the diameters of single arterioles and capillaries at different depths in the brain cortex are measured (Tiana et al., 2010; Devor et al., 2011; Kasischke et al., 2011; Schwarzmaier et al., 2016). As for calcium flux, the astrocytes play a key role, when oxygen is limited after BBB breakdown, the astrocyte glycolysis and lactate release are maximized. The astrocytes induce vasodilation relies on the metabolic state (Gordon et al., 2008). The other cell type is pericyte, by using pericyte-deficient mice, Kisler et al. (2017) show that the pericyte degeneration diminishes capillary CBF responses, resulting in oxygen supply reduction to the brain and metabolic stress.

During primary injuries phase of TBI, the immediate cell death can cause noxious substances release and BBB breakdown. It is confirmed that ROS, mainly generated in the neurons under the pathological conditions, are the key mediators of BBB breakdown, and overproduced after BBB dysfunction (Gilgun-Sherki et al., 2002; Pun et al., 2009). ROS directly downregulate the proteins of tight junctions and indirectly activate MMPs, which lead to leakiness of the BBB and progression of neuroinflammation (Abdul-Muneer et al., 2015). In addition, ROS contribute to active Src family kinases, resulting in further dysfunction of BBB and brain edema (Liu et al., 2016). In addition, Lutton et al. (2017) reported that following TBI, with the BBB hyperpermeability, endothelial activation results in an increase expression of ICAM-1, which induce more ROS generation. Moreover, the excessive glutamate facilitates the excessive calcium influx further results in the generation of ROS, mitochondrial dysfunction, and cell death (Khatri et al., 2018).

### Inflammatory Molecules Accumulation

The inflammatory response starts within hours after initial insult, corresponding with BBB disruption. The animal studies showed that the peripheral neutrophils, macrophages, T cells, and natural killer cells present in the brain within few hours after TBI (Holmin et al., 1998; Holmin and Mathiesen, 2000; Lin et al., 2017). Then, the leukocytes release pro-inflammatory cytokines and then active resident microglia (Schwarzmaier et al., 2013; Cunningham et al., 2014; Schwarzmaier and Plesnila, 2014; Corps et al., 2015; Salvador et al., 2015; Corrigan et al., 2016). Microglia sense a large repertoire of exogenous and endogenous signals and express certain surface and cytoplasmic receptors as a result of activation (Loane and Kumar, 2016).

In the acute phase following TBI, the damaged neurons and other cells release danger-associated molecular patterns (DAMPs) and pathogen-associated molecular patterns (PAMPs) into the brain (Hanisch and Kettenmann, 2007). Microglia response to these environmental signals and change their phenotypes into M1 or M2 (Xu et al., 2017). M1-like phenotype causes neuroinflammation by releasing the high level of pro-inflammatory molecules [tumor necrosis factor-alpha (TNF-α), interleukin-6 (IL-6), IL-12, and IL-1β], chemokines (monocyte chemoattractant protein-1 (MCP-1), CXCL10) into the microenvironment of the brain (Semple et al., 2010; Clausen et al., 2011; Tian et al., 2016; Sanchis et al., 2020; Sen et al., 2020; Zhao et al., 2020). In the lipopolysaccharide-stimulated (LPS) model, microglia are activated and release TNF-α contributed to BBB dysfunction (Nishioku et al., 2010; Semple et al., 2010; Willis et al., 2020). In addition, another study from Schlegel and Waschke (2009) suggested that TNF-α can induce microvascular endothelial barrier breakdown and reduce BBB stabilization by inhibiting cAMP level and Rac1 signaling (Baumer et al., 2009). For M2-like phenotype microglia, it is associated with the memory immune responses and may have either pro- or anti-inflammatory function. They not only produce anti-inflammatory cytokines, such as IL-10, IL-4, and IL-13, but also upregulate several factors, such as Arg1, YM1, FIZZ1, and MRC1 (Ansari, 2015). In chronic phase, inflammation following BBB dysfunction in TBI can be simultaneously helpful and deleterious (Simon et al., 2017). The experiments in the TBI animal models have shown that the levels of IL-1β, IL-6, CXCL8, IL-10, and TNFα are chronically increased together with chronic microglial activation which link to neurodegeneration and dementia, suggesting that the inflammatory molecules accumulation in brain microenvironment following TBI may last for a long time. For apoptotic factors, a study indicated that, following TBI and BBB breakdown, accumulation of caspase-3, an apoptotic factor, and its cleaved tau may contribute to microvascular disruption and cause further chronic BBB damage. This process may also accompanied by the chronic inflammatory responses, such as astrocytes and microglia activation (Glushakova et al., 2017).

## Modulation of Microenvironment for Post-Traumatic Brain Injury Recovery

This section discusses the interventions that have been recently reported to modulate the microenvironment for post TBI recovery. In a neuropathological condition, the microenvironment in the brain can be toxic, which may prohibit the neural recovery. Thus, creating an optimal microenvironment in toxic “soil,” is capable of executing neural repair to promote the post-TBI recovery.

### Eliminate the Toxic Substances and Excessive Water in Microenvironment

The acute microenvironmental changes post-TBI present an attractive target for modulation of the TBI symptoms and the development of cognitive changes later in life. For toxic substances eliminate, the methods should be use of specific receptor inhibitors or prevent the entry of ions, such as sodium and calcium, or reduce the content of toxic substance, e.g., ROS, malondialdehyde (MDA), or glutamate. The water elimination, the widely used mannitol is an osmotic agent, however, only for symptomatic treatment but not causal treatment. More strategies are urgently needed to point at causal treatment of edema to enhance brain microenvironment for recovery.

The administration of many drugs targets different type of toxic substances to enhance the microenvironment for neurological function improvement. The accumulating studies have shown that by inhibiting specific receptors which abundantly expressed in CNS, e.g., arginine-vasopressin (AVP) receptor, bradykinin 2 receptor, β2 adrenergic receptor, endothelin receptors B (ETB), myosin light-chain kinase (MLCK), and peroxisome proliferator-activated receptor γ (PPARγ), brain edema can be reduced (Marmarou et al., 2005; Zweckberger and Plesnila, 2009; Zlotnik et al., 2012; Rossi et al., 2013; Krieg et al., 2015, 2016; Michinaga et al., 2018, 2020; Deng et al., 2020; Table 1). The studies have reported that by using AVP V1 and V2 receptor antagonist, brain water content, and intracranial pressure of CCI model were reduced (Krieg et al., 2015, 2016). Additionally, the bradykinin and its B2 receptors play key roles in TBI recovery (Marmarou et al., 2005; Zweckberger and Plesnila, 2009; Trabold et al., 2010). The other study demonstrated that propranolol and metoprolol, β2 adrenergic receptor inhibitors, reduce excess brain glutamate levels in the microenvironment after TBI (Zlotnik et al., 2012). The highly expressed endothelin-1 (ET-1) in brain after TBI usually links with the BBB dysfunction and increases the inflammatory cytokines and chemokines. It is demonstrated that inhibitory of ETB receptor could reduce the brain edema by decreasing the level of claudin-5, occludin, and zonula occludens-1 proteins (Michinaga et al., 2018). In addition, using a MLCK inhibitor ML-7, cerebral edema can be attenuated in a close head injury model (Rossi et al., 2013). Several drugs which already approved in clinical practice show curative effect in TBI treatment, e.g., pioglitazone, bumetanide, and glibenclamide (Deng et al., 2020; Sawant-Pokam et al., 2020; Jha et al., 2021). However, the mechanism of these drugs for treating TBI only explored in the animal models: Deng et al. (2020) demonstrated that pioglitazone increased the expression of PPARγ after TBI, thus, to alleviate TBI-caused brain edema. To block the water or ion channels is also an option to reduce the brain edema. Inhibition of NKCC1/KCC2 channel (Sawant-Pokam et al., 2020), Sur1-Trpm4 channel (Jha et al., 2021), AQP4 transporter (Farr et al., 2019; Glober et al., 2019), ASIC (Yin et al., 2013), NHE-1 (Zhao et al., 2008).

**TABLE 1:** The pharmacologic agents targeting toxic substances and edema in the microenvironment.

Agents	Target	Main function	Stage	References
V1880	AVP V1	Reduce edema, improve outcome	Preclinical	Krieg et al., 2016
SR 49059/SR-121463A	Vasopressin V1a/V2 receptor	Decrease brain edema	Preclinical	Krieg et al., 2015
Anatibant (LF16-0687)	Bradykinin B2 receptor	Reduce brain edema and ICP	Preclinical	Zweckberger and Plesnila, 2009
Anatibant (LF16-0687Ms)	Bradykinin B2 receptor	Reduce ICP, improve functional outcome	Clinical	Marmarou et al., 2005
Propranolol/Metoprolol	β2 adrenergic receptors	Reduce blood glutamate levels	Preclinical	Zlotnik et al., 2012
BQ788	ET_B_	ET_B_ antagonist, decreases brain edema	Preclinical	Michinaga et al., 2018
ML-7	MLCK	Inhibit MLCK, reduce edema	Preclinical	Rossi et al., 2013
Pioglitazone	PPARγ	Reduce brain edema	Clinical/Preclinical	Deng et al., 2020
Bumetanide	NKCC1/KCC2	Reduce brain edema	Clinical/Preclinical	Sawant-Pokam et al., 2020
Glibenclamide	Sur1-Trpm4	Reduce edema, improve functional outcome	Clinical/Preclinical	Jha et al., 2021
Bicarbonate	ASIC	Reduced edema and functional deficits	Preclinical	Yin et al., 2013
KB-R7943	NHE-1	Reduce edema	Preclinical	Zhao et al., 2008
Acetazolamide	AQP4	Reduce edema	Preclinical	Glober et al., 2019
Poloxamer 188	Plasmalemma	Attenuate TBI-induced brain edema, regulate AQP mRNA expression	Preclinical	Bao et al., 2012
Exendin-4	Glucagon-like peptide-1 receptor	Attenuate genes expressions related with dementia	Preclinical	Tweedie et al., 2016
Lactadherin	Unknown	Reduce cerebral edema, promote microvesicle clearance	Preclinical	Zhou et al., 2018
Ghrelin	Unknown (multiple potential)	Decreases the expression of AQP4	Preclinical	Lopez et al., 2012
Ethanol	Unknown	Reduce AQP mRNA	Preclinical	Wang et al., 2013
DHA	Nrf2 signaling pathway	Decrease ROS and NOX_2_	Preclinical	Zhu et al., 2020
Guanosine	Glutamine synthetase	Suppress glutamate uptake, decrease ROS Production and Na^+^/K^+^-ATPase activity	Preclinical	Gerbatin et al., 2017
DAPT (Notch inhibitor)	Notch pathway	Decrease NOX_2_ and ROS level	Preclinical	Zhang H. M. et al., 2018
L-733,060	NK1R	Inhibit NK1R and release of cytochrome c, reduce ROS	Preclinical	Li et al., 2019
ω-3 PUFAs	Unknown (multiple potential)	Inhibit ROS expression	Preclinical	Ren et al., 2017
Catalase	ICAM-1	Reduce ROS	Preclinical	Lutton et al., 2017

Besides applying the specific receptor inhibitors, some agents may have effects on regulating the essential gene expressions to help eliminate excess water, although the particular target of some agents remains unclear. For instance, poloxamer 188 could attenuate TBI-induced brain edema by regulating AQP mRNA expression (Bao et al., 2012). As an agonist of G-protein coupled receptor (GLP-1R), exendin-4 was confirmed beneficial to both type 2 diabetes mellitus (T2DM) and TBI (Tweedie et al., 2016). The studies report that exendin-4 is able to regulate the gene expression which is associated with TBI-caused dementia (Tweedie et al., 2016). Although there is no evidence that shows the specific target of lactadherin, ghrelin, and ethanol in treating TBI, these agents could influence the brain edema or the expression of AQP4 post TBI (Lopez et al., 2012; Wang et al., 2013; Zhou et al., 2018). To eliminate the toxic substance in brain parenchyma following TBI, the main option is to reduce the content of ROS. There are several agents or molecules that have confirmed to decrease the level of ROS after TBI, e.g., docosahexaenoic acid (DHA), guanosine, dual antiplatelet therapy (DAPT), omega-3 polyunsaturated fatty acids (ω-3 PUFAs), L-733,060, and catalase (Gerbatin et al., 2017; Lutton et al., 2017; Ren et al., 2017; Zhang H. M. et al., 2018; Li et al., 2019; Zhu et al., 2020). Some of these factors may have other functions. For instance, guanosine could suppress the glutamate uptake and decrease Na^+^/K^+^-ATPase activity. By inhibiting tachykinin neurokinin-1 receptor (NK1R), L-733,060 could reduce the release of cytochrome c (Li et al., 2019; Table 1).

### Anti-inflammation to Enhance the Microenvironment

**FIGURE 3:**
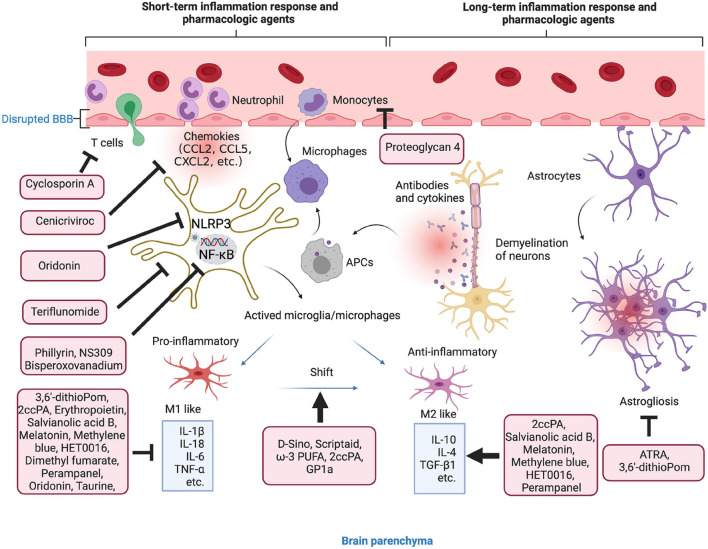
Anti-inflammation strategies in microenvironment. Short- and long-term inflammation response and pharmacologic agents in TBI. The agents in the red boxes showed anti-inflammatory effect in the different stages of inflammatory response.

As mentioned previously, inflammatory response after TBI occurs within minutes and may last for days, weeks, months, or years. Due to the complexity of neural inflammatory response after TBI, certain anti-inflammatory agents are failed to improve the TBI outcomes in some clinical trials (Gaab et al., 1994; Marshall et al., 1998; Asehnoune et al., 2014). For instance, treatment with dexamethasone is failed to improve the Modified Glasgow Coma Scale for the patients with TBI (Gaab et al., 1994). A low-dose of hydrocortisone and fludrocortisone have no effect on the outcome of patients with severe TBI (Asehnoune et al., 2014). However, the emerging pre-clinical studies have been focused on the agents and drugs that can directly target the environmental inflammasome, cytokines, or chemokines, some of them may also alternatively change the macrophage/microglia polarization or regulate classical NF-κB pathway (Table 2 and Figure 3).

**TABLE 2:** The pharmacologic agents with anti-inflammatory effect in the microenvironment.

Agents	Target	Main function	Stage	References
Teriflunomide	DHODH	Inhibit microglia accumulation	Preclinical	Prabhakara et al., 2018
ATRA	Unknown	Protect against astrogliosis and axonal injury	Preclinical	Hummel et al., 2020
D-Sino	Microglia/macrophages	Shift macrophage/microglia polarization toward M2	Preclinical	Sharma et al., 2020
Proteoglycan 4	TLR2/4 and CD44	Curtail the post-traumatic influx of monocytes	Preclinical	Bennett et al., 2021
Scriptaid	HDAC	Shift microglia/macrophage polarization to M2	Preclinical	Wang et al., 2015
3,6′-dithioPom	TNF-α	Lower TNF-α levels, ameliorate astrogliosis	Preclinical	Lin C. T. et al., 2020
ω-3 PUFA	SIRT1	Shift from the M1 microglial phenotype to the M2	Preclinical	Chen X. et al., 2018
2ccPA	Autotaxin	Reduce Iba1 level, suppress IL-1β, IL-6, TNF-α and TNF-β1, increase M2 phenotype	Preclinical	Hashimoto et al., 2018
Cyclosporin A	mPTP	Reduces T-cell counts and activation	Clinical	Chen L. et al., 2020
GP1a (CB2R agonist)	CB2R	Attenuate pro-inflammatory M1 macrophage polarization, increase anti-inflammatory M2 polarization	Preclinical	Braun et al., 2018
Erythropoietin	IL-1 and TNF block erythropoietin production	Increase favorable outcomes without increasing complications	Clinical	Robertson et al., 2014
Phillyrin	PPARγ	Inhibit the proinflammatory response, suppress NF-κB in microglia	Preclinical	Jiang et al., 2020
Bisperoxovanadium	PTEN	Inhibit MCP-1 and AKT/NF-κB p65 pathway	Preclinical	Liu et al., 2019
Salvianolic acid B	Unknown (multiple potential)	Suppress TNF-α and IL-1β, enhance IL-10 and TGF-β1	Preclinical	Chen et al., 2011
Taurine	Unknown (multiple potential)	Decrease 17 cytokines	Preclinical	Su et al., 2014
Melatonin	Unknown	Decrease levels of IL-6 and TNF-α, Increase IL-10	Preclinical	Dehghan et al., 2018
Cenicriviroc	CCR2/5	Decrease gene expression of CCL5, CCL2, CCL7	Preclinical	Morganti et al., 2016
Methylene blue	Unknown	Attenuate microglial activation, reduce IL-1β, increase IL-10	Preclinical	Fenn et al., 2015
HET0016	20-HETE	Decrease the expression of TNF-α, IL-1β, increase the expression of IL-4, IL-10	Preclinical	Shu et al., 2019
Dimethyl fumarate	NF-κB/Nrf-2 pathway	Reduce IL-1β and TNF-α levels	Preclinical	Casili et al., 2018
Perampanel	AMPAR	Suppresses the level of TNF-α and IL-1β, increase IL-10 and TGF-β1	Preclinical	Chen et al., 2017
Oridonin	NLRP3	Reduce secretion of IL-1β and IL-18	Preclinical	Yan et al., 2020
NS309	Potassium SK Channel	Inhibit NF-κB, decreased pro-inflammatory cytokines	Preclinical	Chen et al., 2019

To exert the anti-inflammatory effect, the agents or molecule may target certain type of immune cells to enhance their function, change the phenotypes, inhibit the secretion of pro-inflammatory factors, or enhance the secretion of anti-inflammatory factors (Table 2). There are several agents attenuate inflammation by inhibiting the accumulation and activation of immune cells, such as microglia, T cells, astrocytes, and monocytes (Prabhakara et al., 2018; Chen Y. et al., 2020; Hummel et al., 2020; Bennett et al., 2021). More studies have focused on the process of shifting from M1 microglial phenotype to the M2. For instance, scriptaid, a HDAC inhibitor has been found to play a critical role in shifting microglia/macrophage polarization by upregulating glycogen synthase kinase 3 beta (GSK3β) (Wang et al., 2015). The experimental studies demonstrate that small molecule, such as ω-3 PUFA, GP1a (cannabinoid receptor-2 agonist), attenuate pro-inflammatory M1 macrophage polarization, and increased anti-inflammatory M2 polarization *via* virous pathways (Chen et al., 2017; Lin et al., 2017; Braun et al., 2018; Chen X. et al., 2018).

Genes associated with chemotaxis (CCL2, CCL5, and CCL7), cytokine signaling (IL-6, IL-1β, TNF-β1, TNF-α, and IL-10) can be regulated or specifically inhibited by several agents or drugs, such as 3,6′-dithioPom/Pom (Lin C. T. et al., 2020), 2ccPA (Hashimoto et al., 2018), erythropoietin (Robertson et al., 2014), salvianolic acid B (Chen et al., 2011), taurine (Su et al., 2014), melatonin (Dehghan et al., 2018), cenicriviroc (Morganti et al., 2016), methylene blue (Fenn et al., 2015), HET0016 (Shu et al., 2019), dimethyl fumarate (Casili et al., 2018), and perampanel (Chen et al., 2017). The agents exert anti-inflammatory effect mainly by suppressing the pro-inflammatory factors, e.g., TNF-α, IL-1β, and IL-6, while promoting anti-inflammatory factors, e.g., IL-10 and TGF-β1. For mechanisms, NLRP3 inflammasome attracted much attention in recent years. For instance, oridonin suppresses the expression of NLRP3 inflammasome to decrease the secretion of IL-1β and IL-18 (Yan et al., 2020). In addition, small-molecule NLRP3 inflammasome inhibitor, MCC950, reduces neuroinflammation, preserves BBB integrity, alleviates TBI-induced loss of tight junction proteins, and attenuate cell death in a CCI mice model (Xu et al., 2018). Potassium SK Channel Activator NS309 inhibit NF-κB activation and further decreased the levels of pro-inflammatory cytokines and chemokines (Chen et al., 2019).

### Agents in Microenvironment Targeting Blood-Brain Barrier

As we have discussed, BBB breakdown and the associated microvascular hyperpermeability are hallmark features of TBI pathological change. Thus, the agents contributing to the maintenance of BBB integrity may enhance the microenvironment and further exert brain protective function in TBI.

The efforts aimed at modification of molecular components of the BBB, e.g., TJ, AJ, and BM have shown promising therapeutic effect in treating TBI (Table 3). In recent years, various mediators targeting TJ, AJ, and BM proteins has been confirmed to play important roles in BBB repairment following TBI. Cyclosporin A antagonist CsA has been found to attenuate MMP-9 responses and enhances BBB repair in TBI animal model (Main et al., 2018). Other compounds or molecules, such as microRNA-9-5p agomir (Wu et al., 2020), FABP7 (Rui et al., 2019), mdivi-1 (Wu et al., 2018), bosentan (Michinaga et al., 2020), SB-3CT (Jia et al., 2014), also have effect on expression of the BM proteins (mainly MMP-2 and MMP-9) (Table 3). These agents could inhibit the expression of BM proteins to protect against BBB disruption through different signaling pathways. For instance, by targeting Ptch-1, microRNA-9-5p could alleviate BBB disruption though activating the Hedgehog pathway and inhibiting NF-kB/MMP-9 pathway, and further promote the recovery of neurological dysfunction in TBI (Wu et al., 2020). Wang et al. (2016) demonstrated that rhubarb, a traditional Chinese herbal medicine, prevented activation of gp91phox subunit and protect the BBB *via* modulating NADPH oxidase/ROS/ERK/MMP-9 signaling pathway.

**TABLE 3:** The agents in microenvironment targeting BBB components.

Agents	Target	Main function	Stage	References
Bryostatin-1	Protein kinase C	Increase in the tight junction proteins	Preclinical	Lucke-Wold et al., 2015
Cyclosporin A	MMP-9	Decrease the level of MMP-9, enhances BBB repair	Preclinical	Main et al., 2018
P7C3-A20	Endothelial cells	Increased TJ proteins	Preclinical	Vázquez-Rosa et al., 2020
MicroRNA-9-5p agomir	Ptch-1	Inhibit NF-κB/MMP-9 pathway	Preclinical	Wu et al., 2020
FABP7	Caveolin-1	Protect against BBB disruption, inhibit MMP-2/9	Preclinical	Rui et al., 2019
Mdivi-1	Drp1	Inhibit the expression of MMP-9	Preclinical	Wu et al., 2018
Bosentan	ET-1	ET antagonists, reduces BBB alter the expression of MMP-9	Preclinical	Michinaga et al., 2020
Proteoglycan 4	TLR2/4 and CD44	Prevent the post-traumatic loss of tight junction protein claudin 5	Preclinical	Bennett et al., 2021
rhFGF21	FGFR1/β-klotho complex	Upregulate TJ and AJ proteins	Preclinical	Chen J. et al., 2018
Sesamin	Unk (multiple potential)	Alleviate loss of the TJ proteins	Preclinical	Liu et al., 2017
Capsazepine	TRPV1	Decreases loss of TJ proteins	Preclinical	Yang D. X. et al., 2019
Glibenclamide	JNK/c-jun signaling pathway	Elevate TJ protein expression	Preclinical	Xu et al., 2017
SB-3CT	MMP-9	Inhibit MMP-9	Preclinical	Jia et al., 2014
TIMP1	CD63/integrin β1 complex	Enhance endothelial structure stability	Preclinical	Tang et al., 2020
TIMP3	Endothelial cells	Promotes AJ stability	Preclinical	Menge et al., 2012
Rhubarb	gp91^phox^ subunit	Protect BBB by inhibiting NADPH oxidase/ROS/ERK/MMP-9 pathway	Preclinical	Wang et al., 2016

Besides to suppress the BM proteins, TJ and AJ proteins are also considered as main targets for BBB protection following TBI. A study has demonstrated that P7C3-A20, a compound that stabilizes the cellular energy levels, could increase the expression of TJ proteins in different region of the brain, e.g., claudin-5 in the cortex and hippocampus, and zona occludens-1 in the cortex (Vázquez-Rosa et al., 2020). Other agents or drugs, such as proteoglycan 4, rhFGF21 (Bennett et al., 2021), sesamin (Liu et al., 2017), capsazepine (TRPV1 inhibitor) (Yang D. X. et al., 2019), glibenclamide (Xu et al., 2017), TIMP1 (Tang et al., 2020), and TIMP3 (Menge et al., 2012) also have the effect on the expression of TJ and AJ proteins, such as claudin 5, occludens-1, and ZO-1.

### Molecules and Factors in Microenvironment for Neurogenesis

In a neuropathological condition, the damaged brain can activate a system of self-repair by promoting neurogenesis. Although brain tissue is poor at self-regeneration, in some cases, the quiescent cells can be mitotically activated by the vinous factors in the microenvironment. Recently, the emerging pre-clinical studies have investigated that stem cell transplantation is a novel method for treatment of TBI (Richardson et al., 2010; Koliatsos et al., 2015). However, this therapy has very low rates of cell survival due to the unbefitting microenvironment (Riess et al., 2002). Thus, targeting the specific molecules and factors to enhance the neuro-microenvironment considered to be the strategy. Recent studies show that numerous secrete factors can promote the endogenous repair response, i.e., chemokine stromal cell-derived factor 1α (SDF-1α) (Addington et al., 2015), cytokine signaling-2 (SOCS2) (Basrai et al., 2016), carbon monoxide (Choi et al., 2016), brain-derived neurotrophic factor (BDNF) (Failla et al., 2015; Shi et al., 2016), fibroblast growth factor (FGF2) (Nichols et al., 2013), and Wnt3a (Zhao Y. et al., 2016; Table 4). A new study reported that repopulating microglia can promote brain repair after TBI by regulating IL-6 and IL-6 receptor to support neurogenesis (Willis et al., 2020). In addition, mild hypothermia (MHT) therapy mitigates the degree of microenvironment and benefit for neurogenesis (Chen et al., 2016).

**TABLE 4:** The molecules and factors in the microenvironment for neurogenesis.

Molecules/Factor	Target	Main function	Stage	References
Diazepam	GABA_A_ receptors	Block aberrant post-traumatic neurogenesis	Preclinical	Villasana et al., 2019
Thyroid hormone (T3)	Multiple cells	Promoted adult neurogenesis via neuron–NSC crosstalk	Preclinical	Lin C. et al., 2020
Thioperamide	Histamine H3 receptor	Promote neurogenesis	Preclinical	Liao et al., 2019
7,8-dihydroxyflavone (BDNF mimic)	Multiple cells	Increase the number of adult-born immature neurons	Preclinical	Zhao S. et al., 2016
Cerebrolysin	GABA_B_ receptors	Reduce astrogliosis and axonal injury and promote neurogenesis	Preclinical	Zhang et al., 2019
Wnt3a	Wnt/β-catenin pathway	Increase neurotrophins and regenerative activities	Preclinical	Zhang J. Y. et al., 2018
Neurotrophin-3	Multiple cells	Pro-neurogenesis	Preclinical	Hao et al., 2017
IL-6	IL-6 trans-signaling	Repopulate microglia, modulate the microenvironment	Preclinical	Willis et al., 2020
MSC-FGF21	Multiple cells	Improve impaired hippocampal neurogenesis	Preclinical	Shahror et al., 2020
MSC-generated exosomes	Unknown	Increase the number of newly generated endothelial cells	Preclinical	Zhang Y. et al., 2015
Exo-miR-124	TLR4	Promote the M2 polarization, enhance neurogenesis in hippocampus	Preclinical	Yang Y. et al., 2019
miR-216-5p	HMGB1	Inhibit cell apoptosis and promote neuron regeneration	Preclinical	Xu et al., 2020

Other pathways to enhance the microenvironment for neurogenesis is exosomes delivery (Lai et al., 2013; Zhang et al., 2016). The exosomes are kind of vesicles that carry proteins and RNAs for intercellular communication, and usually have ability to cross the BBB and reach the brain parenchyma. Among them, MSCs-derived exosomes might play an essential role in neurogenesis following TBI and promise to be a novel and valuable therapeutic strategy (Xiong et al., 2017; Yang et al., 2017; Chen Y. et al., 2020). The injection of exosomes derived from the MSCs effectively improve functional recovery after TBI (Zhang Y. et al., 2015). However, the cellular and molecular mechanism of this neurogenic process remains unclear. The majority of the studies are inclined to believe that the MSCs participate in neurogenesis after TBI is not their cell replacement effects but their secretion-based paracrine effect (Zhang et al., 2016). The exosomes-induced microenvironment acts as a crucial role in the regulation of plasticity and homeostasis in the neurogenesis process. The injection of exosomes derived from the MSCs effectively improve functional recovery after TBI. In the recent years, exosomes related studies of TBI focused on miRNAs in exosomes, such as miR-124 and miR-216a-5p (Zhang L. et al., 2015; Yang Y. et al., 2019; Long et al., 2020; Table 4). Moreover, in clinical study, the exosomes can be used as the injury-specific biomarkers for TBI diagnose and considered to be potential therapeutic target (Moyron et al., 2017). Additional emphasis may be placed on promoting endogenous neurogenesis to limit cognitive impairment and to promote repair of the injured brain.

## Conclusion

Traumatic brain injury is a complex, heterogeneous, and mechanobiology problem with the dynamic changes of the microenvironment following BBB disruption (Logsdon et al., 2015; Cash and Theus, 2020). Not only cells and vascular conditions are dramatically changed (Logsdon et al., 2017; Johnson et al., 2018), but also the microenvironment around neurons and other cells. Thus, understanding the underlying mechanisms of these variations after TBI are necessary in appropriate patient management (Lucke-Wold et al., 2015). Abundant studies of brain microenvironment have emerged in the areas of brain tumors and cancers (Subramani et al., 2013; Batista et al., 2015; Placone et al., 2016). However, the evidence of microenvironmental changes following TBI is inadequate. In this review, we briefly overviewed the structure and function of BBB, the pathophysiologic process of microenvironmental changes following TBI-induced BBB breakdown, such as CBF alteration, water imbalance, cerebral metabolism imbalance, and the accumulation of inflammatory molecules. By summarizing the current literature, we also listed the potential intervention to target BBB-disruption-related microenvironment for post TBI recovery. The key aspects included are reducing toxic substances and in the intercellular matrix, eliminating excessive water, inhibiting inflammation, protecting BBB components, and promoting neurogenesis. Over the up-coming years, more emerging information on the mechanism of microenvironmental changes following TBI-induced BBB disruption may help in formulating the novel strategies for post-TBI treatment.

## Author Contributions

YH did major work of writing the manuscript. WT made the outline of this review. Both authors agreed to be accountable for the content of the work.

## Conflict of Interest

The authors declare that the research was conducted in the absence of any commercial or financial relationships that could be construed as a potential conflict of interest.

## Publisher’s Note

All claims expressed in this article are solely those of the authors and do not necessarily represent those of their affiliated organizations, or those of the publisher, the editors and the reviewers. Any product that may be evaluated in this article, or claim that may be made by its manufacturer, is not guaranteed or endorsed by the publisher.

## Abbreviations

2ccPA: 2-carba-cyclic phosphatidic acid
20-HETE: 20-hydroxyeicosatetraenoic acid
ω -3 PUFAs: omega-3 polyunsaturated fatty acids
AJ: adherens junction
AMPAR: α-amino -3-hydroxy-5-methyl-4-isoxazole propionate receptor
APCs: antigen-presenting cell
AQPs: aquaporins
Arg1: arginase 1
ASICs: Acid sensing ion channels
ATRA: All-trans retinoic acid
ATP: adenosine triphosphate
AVP V1: arginine-vasopressin V1
BBB: blood-brain barrier
BDNF: brain-derived neurotrophic factor
BM: basement membranes
CB2R: cannabinoid B2 receptors
CBF: cerebral blood flow
CCI: controlled cortex impact
CCL2: chemokine (C-C motif) ligand 2
CCL5: chemokine (C-C motif) ligand 5
CD44: cluster of differentiation 44
CNS: central nerve systems
CXCL2: chemokine (C-X-C motif) ligand 2
DAMPs: danger-associated molecular pattern
DHA: Docosahexaenoic acid
DHODH: dihydroorotate-dehydrogenase
Drp1: dynamin-related protein 1
D-Sino: dendrimer sinomenine
EAAT2: excitatory amino acid transporter 2
ET-1: endothelin-1
ET-A: endothelin receptors A
ET-B: endothelin receptors B
Exo-miR-124: miR-124 enriched exosomes
FGFR1: Fibroblast growth factor receptor
FIZZ1: resistin-like- α
FGF21: Fibroblast growth factor 21
GABA: gamma-aminobutyric acid
GJ: gap junction
GLT-1: glutamate transporter-1
HET0016: N-hydroxy -N-4-butyl-2-methylphenylformamidine
HDACs: histone deacetylases
HMGB1: high-mobility group box 1
ICAM-1: intercellular adhesion molecule 1
ICP: intracranial pressure
IL: interleukin
JAMs: junctional adhesion molecules
JNK: stress activated protein kinase
Mdivi-1: mitochondrial division inhibitor 1
MCP-1: monocyte chemoattractant protein-1
MLCK: myosin light-chain kinase
MMP: matrix metalloproteinases
mPTP: mitochondrial permeability transition pore
MRC1: mannose receptor C-1
MSC: mesenchymal stem cells
NK1R: tachykinin neurokinin-1 receptor
NKCC1/KCC2: Na^+^-K^+^-Cl^–^ cotransporter 1/K^+^-Cl^–^ cotransporter 2
NF- κ B: transcription factors of the nuclear factor kappa B
Nrf2: nuclear factor erythroid-2 related factor 2
PAMPs: pathogen-associated molecular patterns
PbtO2: trial pressure of brain tissue oxygen
PPAR γ: peroxisome proliferator-activated receptor γ
PTEN: Phosphatase and tensin homolog
Ptch-1: Patched 1
ROS: reactive oxygen species
SEMA3A: Semaphorin 3A
SIRT1: sirtuin1
TBI: Traumatic brain injury
TJ: tight junction
TIMP1: Tissue inhibitor of metalloproteinase-1
TIMP3: tissue inhibitor of matrix metalloproteinase-3
TLR2/4: Toll-like receptor 2/4
TNF- α: tumor necrosis factor-alpha
TRPV1: Transient receptor potential vanilloid 1
NVU: neurovascular units
YM1: chitinase 3-like 3
ZO-1: zonula occludens-1.
